# Essential infantile esotropia: postoperative motor outcomes and inferential analysis of strabismus surgery

**DOI:** 10.1186/1471-2415-14-35

**Published:** 2014-03-25

**Authors:** Adriano Magli, Roberta Carelli, Francesco Matarazzo, Dario Bruzzese

**Affiliations:** 1 Department of Ophthalmology, Pediatric Unit, University of Salerno, Salerno, Italy; 2 Department of Ophthalmology, University Federico II, Naples, Italy; 3 Department of Preventive Medical Sciences, Federico II University, Naples, Italy; 4 Gi.Ma Center, Via Mergellina 44, 80100 Naples, Italy

**Keywords:** Essential infantile esotropia, Motor outcomes, Surgical techniques, Long-term follow-up, Inferential analysis

## Abstract

**Background:**

The aim of this retrospective study is a long-term evaluation of postoperative motor outcomes and the inferential analysis of strabismus surgery in infant eyes with essential infantile esotropia.

**Methods:**

576 patients were compatible with the criteria: confirmed EIE diagnosis, angle ≥ 30 pD, absence of associated ocular anomalies, onset by 6 months of age, absence of hyperopia > 3 Diopters, operation before age 4. Preoperative deviation classes (30–40 pD, 41–59 pD, ≥ 60 pD) were established, different types of surgery were performed. Follow-up was conducted for 5 years after surgery. Longitudinal data were analyzed using general linear mixed models stratified according to the class of pre-operative deviation. A random intercept and a random slope with time (in months) was assumed with an unstructured within subject correlation structure for repeated measurements.

**Results:**

In patients with preoperative angle ≤ 40 pD, a significant interaction effect for intervention by time (F_5,155.9_ = 3.56, p = 0.004) and a significant intervention effect (F_5,226.1_ = 6.41, p < 0.001) on residual deviation were observed; only the intervention 5 showed a residual deviation inside the limits of a partial success. In Class 41-59, a significant interaction effect for intervention by time (F_4,166.7_ = 5.16, p = 0.001), intervention (F_4,178.1_ = 2.48, p = 0.046) and time (F_1,174.6_ = 9.99, p = 0.002) on residual deviation were observed; intervention 7 had the highest degree of stability showing an outcome within the range of a partial success. In Class ≥ 60 pD no significant effect for intervention (F_4,213.9_ = 0.74, p = 0.567), time (F_1,169.5_ = 0.33, p = 0.569) or intervention by time (F_4,160.9_ = 1.08, p = 0.368) on residual deviation was observed; intervention 3,6 and 7 resulted in a residual deviation within the range of a partial success.

**Conclusions:**

We suggest, where possible, a two-horizontal muscles approach in small angle EIE, while a multiple muscles surgery in large angle EIE.

## Background

Essential Infantile Esotropia (EIE) typically onsets within the first 6 months of age in absence of central nervous system abnormalities, exhibiting strabismus angle >30 pD, slight hyperopia, medium grade amblyopia, dissociated vertical deviation, latent nystagmus, oblique muscle dysfunction, abduction restriction and excessive adduction, cross-fixation and reduced or absent binocular vision [1-4].

The incidence of EEI is 1%, according to some authors [5-7]; Archer et al. [8] estimate an incidence at around 0.5% while Helveston and co-workers [9] found it nearer to 0.1%.

Ophthalmological and orthoptic examinations are carried out in the strabismus patient mainly in order to: diagnose or rule out the presence of amblyopia, establish strabismus type, evaluate and define deviation angle, analyze sensory state (for unilateral or alternating exclusion, central suppression, anomalous retinal correspondence, VBS) [10] and stereopsis.

Without doubt, EIE therapy is centred on surgery, however surgical intervention is subordinate to several conditions: horizontal deviation stability, estimation of vertical deviation (if any), absence of accommodative factors, presence of alternating fixation (spontaneous or re-educated), sufficient patient cooperation to set up an appropriate operative program.

## Methods

The aim of the study carried out by our Paediatric Ophthalmic Department, is the long-term evaluation of post operative follow-up of EIE patients, operated within the first 4 years of life.

A retrospective review was undertaken of all EIE surgeries encoded in our database performed by the senior author at is private practice and at the University hospital. Charts of 1248 patients with infantile esotropia who underwent different surgical procedures from 1980 through 2007 were reviewed.

We evaluated patient history, clinical presentation, workup, treatment and outcomes. Between one and three pre-surgical examinations were carried out, in order to establish angle stability. Patients were followed up at 3 months, 6 months, 1 year and 5 years after surgery.

The study inclusion criteria are: confirmed EIE diagnosis, angle ≥ 30 pD, absence of associated ocular anomalies, EIE onset by 6 months of age, absence of hyperopia > 3 Diopters.

All children included in our study were alterning preoperatively; surgical intervention was subordinate to the presence of alternating fixation (spontaneous or re-educated). Patients with only one follow-up or without reliable pre and postoperative measurements were excluded. Patients were also excluded if any CNS abnormality was diagnosed.

No surgery was performed after age 4. All patients had never undergone extraocular muscle surgery before our study.

7 types of surgery were performed, identified by numbers 1 to 7 (Table 1). Type 1 surgery (OO rec RM) patients were excluded from the data analysis, due to too small sample for statistical evaluation.

**Table 1:** Different surgical approaches

**Surgery**	**n (%)**	**Age, mean ± std. dev. (min – max) at 1**^**st **^**surgery; years**
**Type 1**: OO rec MR	6	
**Type 2**: OO rec MR + res LR	29 (5.0)	2.8 ± 0.5 (2–4)
**Type 3**: OO rec MR + OO res LR	71 (12.4)	2.6 ± 0.7 (1–4)
**Type 4**: OO rec MR + OO res LR + rec conj	29 (5.0)	2.9 ± 0.6 (2–4)
**Type 5**: OO rec MR + rec-ap IO	19 (3.3)	2.5 ± 0.5 (2–3)
**Type 6**: OO rec MR + OO res LR + rec-ap IO	156 (27.1)	2.9 ± 0.6 (1–4)
**Type 7**: OO rec MR + OO res LR + rec conj + rec-ap IO	272 (47.2)	2.5 ± 0.7 (1–4)
Total	576 (100)	2.7 ± 0.7 (1–4)

Type 1 (OO rec MR) patients were excluded from the data analysis, due to too small sample for statistical evaluation.

Finally, our statistical sample included 576 patients, compatible with all the above mentioned criteria.

Several parameters were estimated both pre-operatively and post-operatively:

Presence/absence of familiarity and systemic anomalies;

Type of esotropia;

Horizontal deviation (estimated after the complete correction of the refractive error when present; the mean deviation between near and distant is measured by Krimsky test, or, when possible, with cover-test);

Deviation variability (variability was defined when the horizontal deviation angle showed a difference ≥ 15 pD between the first, second and third pre-operative visit; patients presenting variability were excluded from the statistical data analysis);

Ductions and versions, including Inferior Oblique (IO), Superior Oblique (SO), Lateral Rectus (LR), Superior Rectus (SR) and Inferior Rectus (IR) muscles hyper/hypo functions and LR Pseudoparalysis;

Hyperopia; Up-shoot (an arbitrary scale from (+) to (++++) was set up, where the greatest number of + indicated the greatest up-shoot; both RE and LE up-shoots were reported, thus bringing to light any differences between the two eyes);

Alphabetical variations (V or A variations), DVD, anomalous head position, nystagmus, cross-fixation and sensory state evaluation.

Complete eye examination was performed, with Visual Acuity (UCVA and BCVA) and Amblyopia assessment. Classification of amblyopia degree is variable among different authors. We consider mild amblyopia as a BCVA greater than 0.2 logMAR, medium grade or moderate amblyopia a BCVA between 0.2 and 0.5 logMAR, severe amblyopia a BCVA lower than 0.5 logMAR.

Follow-ups were carried out at 3 months, 6 months, 1 year, 5 years. All patients were operated under general anaesthesia.

All patients were discharged day after surgery. Patients were treated with antibiotic-corticosteroids eye drops for 10 days following operation. Informed consent was obtained prior to surgery from the parents, after informing of all peri and post-operative risks. All surgical procedures were conducted in accordance with the Helsinki Declaration.

Patients were divided into classes according to degree of deviation, surgical approach and age at surgery.

Regarding deviation, patients were grouped in three classes. Class 1: 30–40 pD, Class 2: 41–59 pD, Class 3: ≥ 60 pD.

Post-operative success was defined by the following criteria: totally successful (0 pD deviation); partially successful (deviation > 0 and ≤ +10 pD or deviation < 0 and ≥ -10 pD), unsuccessful (deviation > + 10 pD and < -10 pD).

Concerning statistical analysis, SPSS software (version 20.0; SPSS Inc., Chicago, IL, USA) and R (version 2.5.0; The R Foundation for Statistical Computing) were used. Analyses included only available data and missing values were not imputed.

Data were summarized as means ± standard deviation for continuous variables and as frequencies (%) for categorical variables.

Longitudinal data were analyzed using general linear mixed models stratified according to the class of pre-operative deviation. A random intercept and a random slope with time (in months) was assumed with an unstructured within subject correlation structure for repeated measurements. All the models included intervention type, time period, and the interaction between intervention and time, and were further adjusted for the following covariates: preoperative deviation (pD), presence of nistagmus (Yes/No),presence of Lateral Recti muscles Hypofunctions (Yes/No), presence of Inferior oblique muscles hyperfunctions (Yes/No), age at intervention (years) and subsequent intervention (Yes/No).

## Results

The sample included 576 patients (249 F and 327 M). Mean age at surgery was 2.7 ± 0.7 (1–4); 68 (11.8%) patients were operated after 3 years of age.

Regarding the sample distribution: 210 patients (36.5%) had a deviation between 30 and 40 pD, 181 (31.4%) between 41 and 59 pD and 185 children (32.1%) had a pre-operative angle ≥ 60 pD. The mean pre-operative deviation was 49.8 ± 13.2 pD, ranging from 30 to 100 pD.

According to surgery type (Table 1) our patients showed greater frequency of type 7 (47.2%) and type 6 (27.1%) interventions while intervention 5 was the least frequent (3.3%), with different distribution in the different deviation classes. Mean age at first surgery varied slightly according to surgical approach.

Table 2 describes the different deviations found during follow-up visits (3 months, 6 months, 1 year and 5 years). 13.0% of patients achieved 1 to 10 pD esotropia at 5 years follow-up; 18.4% of patients had persistent exotropia less than 10 pD and 34.9% had orthotropia. Number of patients under examination decreases during follow-up, due to patient drop-out for various reasons. Frequency distribution of the 576 subjects, classified according to surgery and according to pre-operative deviation (3 types) is shown in Table 3.

**Table 2:** Angle deviation (pD) during follow-up

**Follow-up**		**Ocular alignment (pD)**					
		**< −10**^**∆**^	**From −10**^**∆ **^**to −1**^**∆**^	**0**^**∆**^	**From +1**^**∆ **^**to +10**^**∆**^	**> + 10**^**∆**^	**Missing**
3 months	n (%)	111 (19.3)	150 (26.0)	131 (22.7)	114 (19.8)	64 (11.1)	6 (1.1)
6 months	n (%)	99 (17.2)	144 (25.0)	140 (24.3)	123 (21.4)	59 (10.2)	11 (1.9)
1 year	n (%)	95 (16.5)	137 (23.8)	184 (31.9)	68 (11.8)	74 (12.9)	18 (3.1)
5 years	n (%)	45 (7.8)	106 (18.4)	201 (34.9)	75 (13.0)	40 (7.0)	109 (18.9)

**Table 3:** Distribution of the 576 subjects used in longitudinal analysis classified according to the surgery type and the pre-operative deviation class

	**Class of pre-operative deviation**		
***Type of intervention***	**30-40**	**41-59**	**≥60**
**2**	23 (79.4)	3 (10.3)	3 (10.3)
**3**	27 (38.1)	16 (22.5)	28 (39.4)
**4**	16 (55.2)	6 (20.7)	7 (24.1)
**5**	19 (100.0)	0 (0.0)	0 (0.0)
**6**	76 (48.7)	30 (19.2)	50 (32.1)
**7**	49 (18.0)	126 (46.3)	97 (35.7)
**Total**	210 (36.5)	181 (31.4)	185 (32.1)

Data concerning entity of recessions and resections in terms of mm are reported in Table 4.

**Table 4:** Descriptive analysis of millimeters (mm) of intervention, mean, median and range according to the different surgery types

**Type of intervention**	**mm of intervention**	**RE**	**LE**	**RE**	**LE**	**RE**	**LE**	**RE**	**LE**
		**rec MR**	**rec MR**	**res LR**	**res LR**	**rec conj**	**rec conj**	**rec anter IO**	**rec anter IO**
**2**	Mean ± Std. Dev.	4.22 ± 0.51	4.41 ± 0.57	8 ± 0.53	7.38 ± 0.74				
	Median [min; max]	4 [3; 5]	4 [3; 5]	8 [7; 9]	7 [6; 9]				
**3**	Mean ± Std. Dev.	4.02 ± 0.59	4.01 ± 0.65	6.65 ± 0.86	6.7 ± 0.85				
	Median [min; max]	4 [3; 6]	4 [3; 7]	6 [5; 9]	7 [5; 9]				
**4**	Mean ± Std. Dev.	4.59 ± 0.61	4.53 ± 0.6	7.22 ± 0.64	7.12 ± 1.05	3.83 ± 0.54	3.85 ± 0.53		
	Median [min; max]	5 [3; 5.5]	5 [3; 5.5]	7 [6; 9]	7 [4; 9]	4 [2; 4]	4 [2; 4]		
**5**	Mean ± Std. Dev.	6 ± 0	5.95 ± 0.23					6.95 ± 0.23	6.95 ± 0.23
	Median [min; max]	6 [6; 6]	6 [5; 6]					7 [6; 7]	7 [6; 7]
**6**	Mean ± Std. Dev.	4.38 ± 0.54	4.36 ± 0.52	6.53 ± 0.59	6.47 ± 0.74			6.59 ± 1.13	6.66 ± 0.81
	Median [min; max]	4 [3; 7]	4 [3; 6]	6 [5; 8]	6 [4; 9]			6 [2; 9]	7 [5; 8]
**7**	Mean ± Std. Dev.	4.6 ± 0.57	4.73 ± 0.54	6.81 ± 0.51	6.85 ± 0.55	3.98 ± 0.16	3.98 ± 0.16	6.56 ± 1.23	7.3 ± 0.89
	Median [min; max]	5 [3; 6]	5 [3; 6]	7 [6; 9]	7 [5; 9]	4 [3; 5]	4 [3; 5]	7 [4; 9]	7 [5; 9]

(RE = right eye, LE = left eye, rec = recession, res = resection, rec ant = recession and anteroposition, MR = medial rectus, LR = lateral rectus, conj = conjunctiva, IO = inferior oblique).

**Figure 1:**
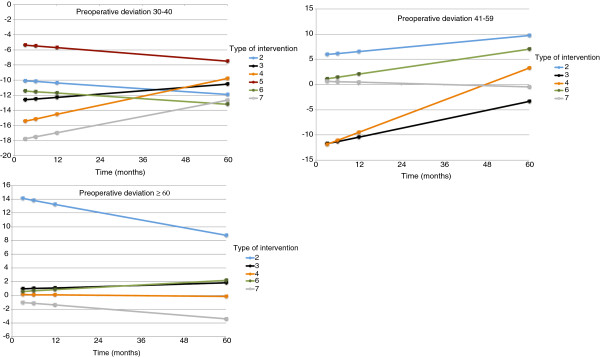
Estimated marginal means (with 95% C.I) of residual deviation during follow-up in the different groups.

Longitudinal analysis. Mean post-operative residual deviations, with 95% confidence intervals (CIs), for each intervention type at 3 months, 6 months, 1 year and 5 years, classified according to the class of pre-operative deviation are presented in Figure 1, Tables 5, 6 and 7.

**Table 5:** **Estimated marginal means (with 95**% **C.I) of residual deviation for all intervention types over time in the class of pre-operative deviation 30 pD – 40 pD**

	**Follow up**			
**Type of intervention**	**3 months**	**6 months**	**1 year**	**5 years**
2	−10.1 [−15.1; −5.1]	−10.2 [−15.1; −5.3]	−10.4 [−15.1; −5.6]	−11.9 [−16.8; −7]
3	−12.6 [−17.5; −7.7]	−12.5 [−17.3; −7.7]	−12.3 [−16.9; −7.6]	−10.5 [−15.8; −5.3]
4	−15.4 [−21.5; −9.4]	−15.1 [−21.1; −9.2]	−14.5 [−20.3; −8.8]	−9.8 [−15.8; −3.7]
5	−5.4 [−10.6; −0.1]	−5.5 [−10.6; −0.3]	−5.7 [−10.7; −0.7]	−7.5 [−12.6; −2.4]
6	−11.5 [−15.5; −7.4]	−11.5 [−15.6; −7.5]	−11.7 [−15.7; −7.7]	−13.2 [−17.2; −9.2]
7	−17.8 [−22; −13.5]	−17.5 [−21.7; −13.3]	−17 [−21.1; −12.8]	−12.7 [−17.2; −8.1]

**Table 6:** **Estimated marginal means (with 95**% **C.I) of residual deviation for all intervention types over time in the class of pre-operative deviation 41-59 pD**

	**Follow up**			
**Type of intervention**	**3 months**	**6 months**	**1 year**	**5 years**
2	6 [−9.8; 21.8]	6.2 [−9.1; 21.4]	6.6 [−7.7; 20.8]	9.7 [−2.1; 21.5]
3	−11.8 [−19.6; −3.9]	−11.3 [−19; −3.6]	−10.4 [−17.7; −3.1]	−3.4 [−9.6; 2.9]
4	−11.9 [−23.5; −0.2]	−11.1 [−22.3; 0.2]	−9.5 [−20; 1]	3.3 [−5.2; 11.8]
6	1.2 [−6.1; 8.4]	1.5 [−5.7; 8.6]	2.1 [−4.8; 9]	7 [0.6; 13.4]
7	0.6 [−5.2; 6.5]	0.6 [−5.2; 6.4]	0.4 [−5.3; 6.2]	−0.5 [−6.1; 5.2]

**Table 7:** **Estimated marginal means (with 95**% **C.I) of residual deviation for all intervention types over time in the class of pre-operative deviation ≥ 60 pD**

	**Follow up**			
**Type of intervention**	**3 months**	**6 months**	**1 year**	**5 years**
2	14.1 [−2; 30.2]	13.8 [−1.8; 29.4]	13.3 [−1.4; 27.9]	8.8 [−4.2; 21.7]
3	1 [−5.2; 7.1]	1 [−5.1; 7.1]	1.1 [−4.7; 6.9]	1.8 [−3.3; 7]
4	0.1 [−10.3; 10.6]	0.1 [−10; 10.2]	0.1 [−9.4; 9.6]	−0.1 [−7.9; 7.6]
6	0.6 [−5.2; 6.4]	0.6 [−5.1; 6.4]	0.8 [−4.7; 6.4]	2.2 [−3; 7.4]
7	−1 [−7.2; 5.2]	−1.1 [−7.3; 5]	−1.4 [−7.5; 4.7]	−3.4 [−9.3; 2.5]

Class 30–40 pD. A significant interaction effect for intervention by time (F_5,155.9_ = 3.56, p = 0.004) and a significant intervention effect (F_5,226.1_ = 6.41, p < 0.001) on residual deviation were observed. No significant effect for time emerged (F_1,155.8_ = 1.9, p = 0.168). The covariate of a second intervention was also observed to have a significant impact on residual deviation (F_1,161.4_ = 80.44, p < 0.001) with an average reduction of 17.4 pD (95% C.I. 13.6 pD; 21.3 pD).

Class 41–59 pD. A significant interaction effect for intervention by time (F_4,166.7_ = 5.16, p = 0.001), intervention (F_4,178.1_ = 2.48, p = 0.046) and time (F_1,174.6_ = 9.99, p = 0.002) on residual deviation were observed. The following covariates also had a significant impact on residual deviation: Nistagmus (F_1,144.4_ = 6.34, p = 0.013) and Lateral Recti Hypofunction (F_1,149.6_ = 28.93, p < 0.001) causing, during 5 years follow up after the intervention, an average increase in the residual deviation of 4.8 pD (95% C.I. 1.1 pD; 8.5 pD) and 7.0 pD (95% C.I. 4.4 dP; 9.5 pD) respectively.

Class ≥ 60 pD. No significant effect for intervention (F_4,213.9_ = 0.74, p = 0.567), time (F_1,169.5_ = 0.33, p = 0.569) or intervention by time (F_4,160.9_ = 1.08, p = 0.368) on residual deviation was observed. The covariate Lateral Recti Hypofunctions was also observed to have a significant impact on residual deviation (F_1,152.8_ = 7.07, p = 0.009) with an average increase of 4.9 pD (95% C.I. 1.3 pD; 8.6 pD).

Concerning reoperations, 57 patients out of 576 (9.9%) underwent a second surgery. Type 5 surgery lead to highest reoperation rate (52% of patients who underwent type 5).

## Discussion

Aims of all EIE surgery are the early or near recovery of orthotropy with the lowest possible number of surgeries.

The traditional surgical approach, maintained by several authors, is medial recti bilateral recession [10-16].

In cases of small deviation angles, majority of authors suggest the double recession of the medial recti, possibly associated with unilateral lateral recti resection.

Optimal surgical technique for large-angle esotropia correction is still controversial. Surgery can be performed on 3 muscles, with double medial recti recession and unilateral lateral rectus resection [17] or on 4 muscles (double medial recti recession with bilateral resection of the lateral recti) [18].

According to Vroman et al., the success rate for ocular realignment in patients with EIE by using bilateral medial rectus muscle recession did not appear to diminish when applied to deviations greater than 50 pD, as compared with smaller angle deviations. In their opinion, surgery on 3 or 4 horizontal rectus muscles may be unnecessary in the treatment of patients with very large angles [19]. Some surgeons prefer a large medial recti recession [20-22]. A seven-millimeter bilateral medial rectus recession is considered by some authors a possible approach in large angle congenital esotropia [23,24], as alternative to multiple muscle procedures in the initial treatment of large-angle EIE, leaving the lateral rectus muscles unoperated for future surgeries, if necessary. Those who support large recession [25,26], such as Helveston and Prieto-Diaz, claim that in their cases [26], this surgical choice led to a reduction in re-interventions number, obtaining eye alignment in over 70% of cases. On the other hand, some authors suggest in large-angle infantile esotropia (≥60 PD) a three horizontal muscle surgery, appearing to have a good long-term success rate without leading to high rates of either residual esotropia or consecutive exotropia [27,28].

In our opinion, in essential infantile esotropia, surgery on fewer muscles might lead to an increase in surgical dosage (supramaximal recessions) with greater incomitances in lateral gaze positions. Therefore, we rarely perform only medial recti bilateral recession (type 1). In our cohort, 6 cases who had undergone this approach, operated before age 4, were fulfilling all the inclusion criteria. Nevertheless these cases were not included in the analysis, due to too small group sample. Accordingly, type 2 and type 5 surgeries have been used by us less frequently, compared to other surgical options.

On the other hand, we believe that a multiple muscles approach can give the opportunity to reduce recessions and distribute them amongst all horizontal recti. A minimal and harmonious dosage may reduce the incidence of incomitances, with excellent results even in large esodeviations. When oblique muscle hyperfunction is also present, it is essential to carry out an inferior oblique muscle recession with anteroposition, in addition to horizontal surgery. No myotomies, myectomies o disinsertions were performed in our cohort for cases with oblique dysfunction.

In order to correct hypertropia, in patients with DVD, a superior rectus recession can also be carried out. In our cohort, entity of DVD, when present, did not require an approach during first surgical procedure.

Furthermore, all types of surgery can also be associated with conjunctival recession.

Our inferential analysis results show the distribution of confidence intervals during follow-up in the different deviation groups. Regarding pre-operative deviations ranging between 30–40 pD, it is seen that during the 5 year follow up, only the intervention 5 showed a residual deviation inside the limits of a partial success while intervention 7 showed the worse results leading to a secondary exotropia. Concerning deviations between 41 and 59 pD, intervention 7 had the highest degree of stability during the 5 year follow up, showing an outcome always within the range of a partial success. Partial success 5 years after surgery was also observed for intervention 3, despite showing exotropia during follow-up. Intervention 6 on the other hand, presented stability during the first year after surgery but was associated with residual esotropia at last follow-up. In patients presenting preoperative angle ≥ 60 pD, during the whole follow-up period, intervention 3,6 and 7 resulted in a residual deviation within the range of a partial success. The covariate Lateral Recti Hypofunctions was also observed to have a significant impact on residual deviation, increasing, when present, the residual deviation.

Thus, our inferential analysis allows us to suggest, in deviations ≤ 40 pD, a surgery performed only on two medial recti muscles (associated in our series with treatment of inferior oblique overaction). On the other hand, in small angle esotropia, an approach on multiple horizontal muscles might lead to an increased risk of postoperative exotropia.

Furthermore, in our data, type 7 surgery was the most valid therapeutic approach in children with a pre-operative deviation ≥ 60 pD (the so-called “large angle esotropia”), showing greater stability of residual ocular deviation results over time.

We consider it essential, therefore, to recommend, where possible, at first surgery in large angle esotropic children, a multiple muscle approach and a less invasive approach in small angle deviations.

Concerning reoperations, 9.9% of patients underwent re-operation. The frequency of re-intervention on patients operated with type 5 is higher, relatively to operation type. This suggests that an approach including only MR and IO, without LR, might increase re-operative rate. Nevertheless these data are considered preliminary data and should be further analyzed in a statistical model.

Another important result concerns the importance of follow-up. In the past, authors suggested that residual deviation results, detectable in later stages, could be foreseen at 6 weeks follow-up. In a recent study, the alignment at distance was recorded with a mean of 10.9 years after first surgery, forty-five percent of patients being within 8Δ of orthotropia [29]. Previous studies have reported higher rates of postoperative microtropia, with shorter postoperative durations [30,31]. Reports with longer follow-up periods have fewer microtropic patients at the final follow-up examination. This finding underscore the importance of longterm follow-up, in order to define the evolution of congenital esotropia after surgical intervention.

Our study amply demonstrates that our data show broad oscillations at different post-operative examinations, even though some values keep within defined intervals, underlining the importance of long-term follow-up.

The main limitations of this study are the scarceness of analyzable data after 5 years postoperatively, the difficulties of comparing data at superimposable biological ages and the absence of analysis concerning visual acuity during follow-up.

## Conclusions

In conclusion, in our study, the most successful surgery for large angle EIE patients, in terms of ocular deviation stability over the long period of observation, is double medial recti recession associated with double lateral recti resection, together with inferior oblique recession and anteroposition. Our results agree with other authors [27,28] suggesting this approach in large angle esotropia, for the low rate of either residual esotropia or consecutive exotropia and the good long-term success rate.

Further studies are necessary to extend our results, overcome the limitations of the study and carry out improvements suggested by the results of other authors.

## Competing interests

The authors declare that they have no competing interests.

## Authors’ contribution

AM and RC planned the study, FM and RC collected the data and drafted the manuscript. DB performed the statistical analysis. AM reviewed the final paper. All authors read and approved the final manuscript.

## Pre-publication history

The pre-publication history for this paper can be accessed here:

http://www.biomedcentral.com/1471-2415/14/35/prepub
